# EXPLORING THE FEASIBILITY OF PLATELET-RICH PLASMA INJECTIONS FOR INTERVERTEBRAL DISCOPATHY: A PILOT STUDY

**DOI:** 10.2340/jrm-cc.v7.18305

**Published:** 2024-10-16

**Authors:** Jean-François KAUX, Christophe DEMOULIN, Marie-Antoinette FERRARA, Robert FONTAINE, Stéphanie GROSDENT, Sarah BETHLEN, Marco TOMASELLA, Philippe GILLET, Marc VANDERTHOMMEN

**Affiliations:** 1 Department of Physical Activity and Rehabilitation Sciences, University of Liège, Liège, Belgium; 2 Physical and Rehabilitation Medicine Department, University Hospital of Liège, Liège, Belgium; 3 Medical Imaging Department, University Hospital of Liège, Liège, Belgium; 4 Anesthesia & Intensive Care Department, University Hospital of Liège, Liège, Belgium; 5 Orthopedic Surgery Department, University Hospital of Liège, Liège, Belgium

**Keywords:** back pain, case series, disability, disc degeneration, injection, platelet-rich plasma

## Abstract

**Objective:**

This longitudinal pilot study aimed to evaluate the feasibility, safety and potential benefits of Platelet-Rich Plasma injections into the lumbar intervertebral discs in patients with low back pain and degenerative intervertebral monodiscopathy, assessing potential efficacy on disability.

**Design:**

Longitudinal pilot study.

**Methods:**

Six participants with chronic low back pain and lumbar degenerative intervertebral disc (monodiscopathy) disease underwent 1 Platelet-Rich Plasma injection, with a 1-year follow-up. Platelet-Rich Plasma injections were administered into the lumbar intervertebral disc, and outcomes were measured using the Roland Morris Disability Questionnaire, numeric rating scale for pain, Tampa scale for kinesiophobia and lumbar flexion range. Magnetic resonance imaging analysis assessed disc changes.

**Results:**

No adverse events were reported. At the end of the 1-year follow-up, half of the patients showed significant improvements in disability scores at 1 year, while 3 of the 6 patients had no change. Magnetic resonance imaging revealed no significant disc changes.

**Conclusion:**

Platelet-Rich Plasma injections show promise for some patients with low back pain and degenerative intervertebral discopathy patients. However, caution is warranted due to study limitations, including small sample size and lack of a control group. Further research is needed to define Platelet-Rich Plasma therapy protocols.

## Lay Abstract

This study investigated the feasibility, safety and potential benefits of Platelet-Rich Plasma injections for patients with back pain having a degenerative intervertebral monodiscopathy. Six participants received Platelet-Rich Plasma injections and were monitored for 1 year. We evaluated their pain levels, daily functioning, fear of movement and range of motion before the injection and at the 1-year follow-up, alongside magnetic resonance imaging scans to assess disc changes. Results showed that while half experienced improved daily functioning, the others showed no change. Magnetic resonance imaging scans revealed no significant disc changes. These findings underscore the need for further research, given the study’s small sample size and lack of a control group. Clarifying the benefits of Platelet-Rich Plasma therapy for degenerative discopathy-related low back pain requires additional investigation to establish definitive treatment guidelines.

Low back pain (LBP) presents a significant global health burden and stands as a primary cause of disability (1). Most LBP may resolve with time, but a substantial proportion of individuals endure persistent pain and disability for more than 3 months (2). The complex nature of chronic LBP, with its multifactorial origins and influence of various bio-psycho-social factors, remains an area of ongoing investigation (3). The intervertebral disc is among the numerous anatomic structures, which might contribute to LBP. Indeed, despite efforts to elucidate the relationship between degenerative intervertebral disc disease (DIVD) and LBP, definitive conclusions remain elusive (4). The challenges in attributing LBP solely to disc pathology were highlighted, particularly as no widely accepted reference standard for discogenic pain exists (3). Recent research underscores the complexity of this relationship and the limitations in definitively identifying disc-related causes of LBP (5, 6). Conservative management approaches may not always provide long-term relief, while surgical interventions carry inherent risks (7–9). Furthermore, the avascular nature of the intervertebral disc poses additional challenges for effective treatment strategies (10). Thus, while advancements in understanding the pathophysiology of LBP continue, the quest for optimal management strategies persists.

Platelet-Rich Plasma (PRP) injection is an innovative therapeutic approach used for various musculoskeletal pathologies (11, 12). The technique involves the extraction of a platelet concentrate from an anticoagulated autologous blood sample. The platelet concentration differs from 1 technique to another depending on the therapeutic technique (13). Platelets contain multiple growth factors and proteins that are associated with tissue repair. Despite debates, there is increasing scientific evidence of its clinical effectiveness, particularly in conditions like patellar tendinopathy and epicondylitis (14). PRP has shown promise in improving pain and function in patients with knee osteoarthritis (15). However, a scientific gap exists due to variations in PRP preparation protocols and limited standardization of injection techniques (13, 16, 17). Better classification and understanding of PRP characteristics based on therapeutic indications are needed (18).

In both animal and human studies, PRP has demonstrated potential in stimulating intervertebral disc regeneration by interacting with the extracellular matrix (19). However, research on PRP’s effectiveness in relieving pain has produced inconsistent results.

This pilot study explored PRP injections as a treatment for LBP in patients with DIVD, focusing on feasibility and safety rather than definitive conclusions on effectiveness. Clinical evaluations and medical imaging aimed to understand PRP’s potential role in treating DIVD-related LBP.

## METHODS

This longitudinal clinical study, approved by the Faculty Ethics Committee of the University and University Hospital of Liège (number B707201525421 – 2015/191), focused on patients with chronic LBP.

Patients were enrolled during consultations in the departments of Physical and Rehabilitation Medicine or Neurosurgery for chronic LBP at the University Hospital of Liège (Belgium) following their voluntary informed consent.

Our inclusion criteria were as follows:

- Age between 18 and 60 years to ensure sample homogeneity within an age group commonly affected by lumbar disc diseases.
- Presence of signs and symptoms consistent with lumbar discopathy, including localized LBP, pain exacerbated by specific movements, tenderness over affected disc level, limited range of motion and absence of nerve root irritation signs. Patients with non-mechanical LBP or signs of sensitization were excluded.
- Duration of LBP over 6 months (persistent symptoms).
- Evidence of degeneration in a single DIVD (exclusion if more than 1 lumbar level) confirmed by MRI findings consistent with disc degeneration, bulging, Modic changes or other degenerative changes.
- Patient with systemic or localized infection, symptomatic stenosis, pregnancy, radicular pain, allergies, heavy narcotic use, disc herniation, disc space narrowing over 50%, scoliosis or spondylolisthesis and contraindications to PRP treatment (platelet concentration <50,000/microliter, active infection or cancer) were excluded to minimize confounding factors and ensure participant safety.

Details regarding their characteristics are available in Table I. To evaluate physical activity levels, we directly asked participants about their engagement in physical activity on a weekly basis.

**Table I:** Patients’ information. Physically active = engaging in regular physical activity

	Gender (M/F)	Age (years)	Physically active (Y/N)	Pain duration (years)	Degenerated disk	PRP injected volume (mL)
**Patient 1**	M	44	Y	10	L4–L5	3
**Patient 2**	M	45	N	3	L4–L5	3
**Patient 3**	M	34	N	2	L3–L4	2.5
**Patient 4**	M	38	Y	8	L4–L5	3
**Patient 5**	F	58	N	15	L4–L5	3.5
**Patient 6**	F	40	Y	3	L4–L5	2.5

### Intervention

In this 1-year longitudinal study, patients attended 2 assessments sessions i.e., at baseline and at 12 months after injection, collecting side effects via patient reporting.

### Assessment

Recommended scales were used to assess various parameters. Changes at the 1-year follow up were compared to minimal clinically important difference (MCID) to analyse the clinical importance of the changes. Mean and SD were used for paired t-tests comparing pre-injection and 1-year follow-up results, aiming to detect statistically significant differences (p<0.05).

The primary outcome was disability, measured using the Roland-Morris Disability Questionnaire (RMDQ). Secondary outcomes included pain intensity, assessed with the Numeric Rating Scale (NRS), kinesiophobia, evaluated using the Tampa Scale for Kinesiophobia (TSK) and lumbar flexion, measured with inclinometers. Additionally, MRI was used to analyse disc changes.

- The RMDQ, a 24-question questionnaire with a maximum score of 24 points, was used to assess disability in LBP. A 3-point MCID was considered clinically significant for measuring improvements or deteriorations in LBP disability (20).
- Pain intensity in the lower back over the past 7 days was measured using the NRS on a scale of 0–10 (21). A MCID of 2 points on the NRS scale was deemed clinically significant for detecting changes in pain intensity.
- The TSK, a 17-item questionnaire with scores ranging from 17 to 68, was used to assess Kinesiophobia (22). An MCID of 6 points on the TSK scale was considered clinically significant in assessing changes in kinesiophobia levels.

Lumbar mobility, specifically during maximum trunk flexion with knee extension in standing, was measured using inclinometers (23). An MCID of 10 degrees for lumbar mobility was considered clinically significant in assessing improvements or deteriorations in this aspect.

- MRI scans were interpreted by a skilled musculoskeletal radiologist using established diagnostic criteria for assessing degenerative changes in the lumbar spine. Criteria included evaluation of disc morphology, signal intensity, disc height, presence of bulging or herniation, Modic changes and other structural abnormalities based on established guidelines (24).

### PRP sampling technique

The Dual Needle Thrombopheresis (PLT5d DN) program was used to separate blood cells in this study. The procedure involved utilizing a blood cell separator (COM.TEC) with a unique C5L separation chamber from Fresenius-Kabi, which featured continuous spirals with an integrated barrier (25).

The platelet (PLT) separation process was automated, with a charge-coupled device camera monitoring separation. Plasma flow rate adjusted for deviations. Centrifugation speed set at 2200 rpm, producing 578 times g. The interface was set to 31, and the endpoint was automated based on platelet yield to ensure optimal concentration. The device computed various parameters, including blood flow rate, anticoagulant flow rate, the ratio of blood to anticoagulant and the duration of the process, to maintain accuracy and consistency. Patients were instructed to fast for 3 h before the procedure to prevent micelle formation, which could interfere with the quality of the PRP. To ensure traceability, each sample was meticulously tracked from collection to processing and application.

*Preparation of the apheresis machine.* The C5L apheresis kit and chamber were set up as per the manufacturer’s instructions. NaCl and ACD solutions were connected, and priming displaced air. COM.TEC system detected and matched procedures, monitoring alarms for machine functionality.

*PRP collection.* Patients, positioned supine, connected to apheresis machine via closed dual circuit with venous catheters. Machine settings were based on anthropometric and biologic values of the patient to calculate time for standardized PRP collection (850,000 platelets/microliter) (26). Separated PRP was collected in a separate bag for platelet storage; other components were re-infused.

### Intra-discal PRP injection

**Fig. 1:**
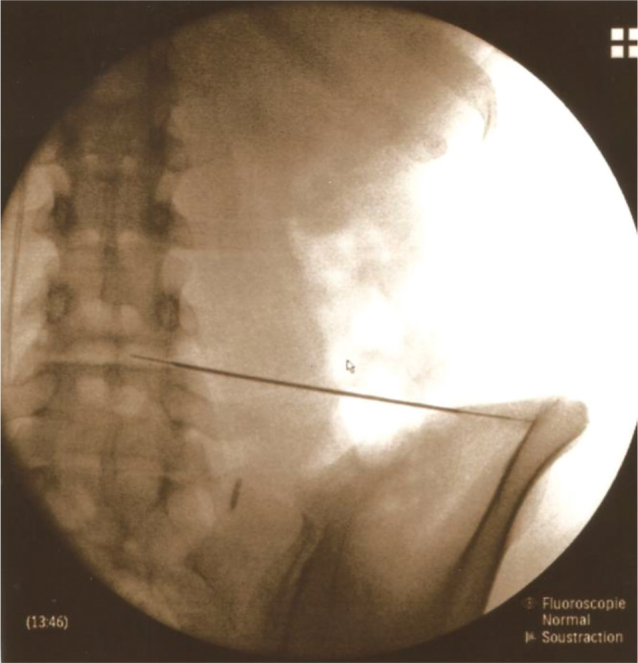
Placement of the needle for an PRP injection within the L4–L5 disc under radioscopic control.

PRP injections were administered in the day hospital’s operating room by an experienced anaesthetist in discography, with the patient under local anaesthesia and in prone position. PRP, homogenized before collection, was withdrawn using a 10 mL syringe (containing 0.1 mL of NaHCO_3_) with an 18 g trocar. Platelets were activated by adding calcium chloride. Injection under radioscopic guidance ensured accuracy (Fig. 1). PRP volume varied (2.5–3.5 mL) based on pain tolerance. Post-injection, patients were monitored for anaesthesia-related complications for at least 1 h in the hospital and provided with level 2 analgesic treatment.

## RESULTS

In this pilot study, 6 patients participated after giving informed consent. Table I summarizes baseline characteristics and demographic details.

**Fig. 2:**
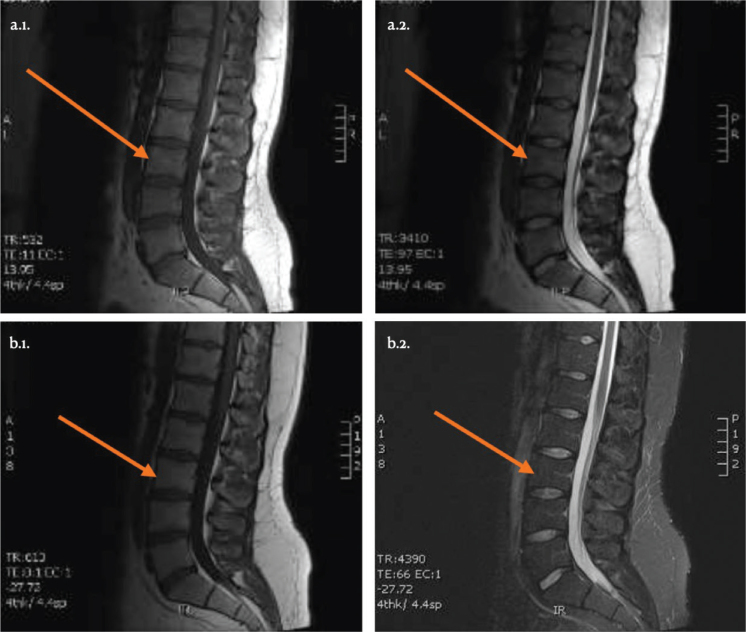
MRI sagittal sections of patient 3, before PRP injection (a.1. T1 sequence; a.2. T2 sequence) and at 12 months post-injection (b.1. T1 sequence; b.2. T2 sequence).

In the 1-year follow-up, no adverse events were reported. Table II shows an improvement in 50% of the participants’ conditions, with notable pain reduction. The responders were 3 men aged 34 to 45 (patients 2, 3 and 4) who experienced significant improvements in RMDQ scores and pain levels above MCID. Although not all patients exceeded the MCID, the overall pain reduction across all 6 patients was statistically significant (p<0.05). Kinesiophobia and lumbar mobility improved in 1 patient (patient 3), who also resumed physical activity post-injection. No significant MRI changes were observed at the 1-year follow-up (Fig. 2).

**Table II:** Patients’ follow-up

	RMDQ (0–24)			Pain (0–10)			Tampa scale (17–68)			Lumbar flexion (degrees)		
	Pre	1 year	MCID≥3 (Y/N)	Pre	1 year	MCID≥2 (Y/N)	Pre	1 year	MCID≥6 (Y/N)	Pre	1 year	MCID≥10° (Y/N)
Patient 1	11	12	N	5	3	Y	55	44	Y	49	31	N
Patient 2	13	4	Y	6	0	Y	55	54	N	41	31	N
Patient 3	12	2	Y	5	3	Y	36	20	Y	12	30	Y
Patient 4	14	3	Y	7	5	Y	38	36	N	51	45	N
Patient 5	18	16	N	6	5	N	56	53	N	12	26	Y
Patient 6	9	9	N	3	3	N	38	34	N	30	37	N
Mean	12.8	7.7		5.3	3.2		46.3	40.2		32.5	32.5	
SD	2.8	5.6		1.4	1.8		9.9	12.9		17.5	6.7	
*t*-Test	0.067			0.048			0.052			0.892		

RMDQ: Roland-Morris Disability Questionnaire; MCID: minimal clinically important difference.

## DISCUSSION

In this pilot study, we assessed the safety of PRP injection for patients suffering from chronic LBP with a 1 level DIVD. The findings demonstrate that PRP injection was well-tolerated, with no reported adverse events or deterioration in the patients’ algo-functional status at the 1-year follow-up. While these results provide preliminary support for the technique’s feasibility, it is essential to interpret them with caution due to the small sample size and the exploratory nature of the study. Additionally, the use of autologous blood for PRP infiltration may contribute to reducing the risk of complications (27), and its antimicrobial properties render it a potentially safer alternative to corticoid infiltrations (28). Furthermore, its minimally invasive nature and lower cost than surgery (9) add to its appeal.

Our study yielded diverse outcomes at the 1-year follow-up mark. While 50% of the participants exhibited noteworthy enhancements in their disability and pain scores, surpassing the threshold for MCID, the remaining 50% experienced no substantial changes in their disability, pain levels or kinesiophobia. Notably, those participants who demonstrated improvements also resumed physical activity following PRP injection, potentially contributing to their positive outcomes (29). Emphasizing the significance of integrating tailored, progressive rehabilitation or fitness programs alongside PRP injection to potentially enhance outcomes warrants consideration. However, this aspect requires dedicated investigation in future studies to establish its efficacy conclusively.

Comparison with existing literature on lumbar intradiscal PRP injection indicates encouraging but mixed results regarding the improvement of functional disability scores (30). Previous studies have reported varying degrees of improvement, ranging from 47% to more conclusive results with sustained positive outcomes after 1 year (31–33). However, it was noted that few studies have focused on functional disability measured with questionnaires and did not include any clinical assessment of function as the measurement of lumbar mobility following an intradiscal PRP injection. Furthermore, the major criticism of the use of PRP is the lack of characterization of PRP and compliance with MIBO criteria (18).

Interestingly, no changes were seen on MRI at the 1-year follow-up, even in the 3 patients who reported clinical improvements. The multifactorial nature of the pain and the psychosocial component of chronic LBP may be one of the reasons for this (34, 35). The strength of the present pilot study was the particularly comprehensive protocol concerning the profile and evolution of participant analyses, not only of pain, function, kinesiophobia, physical activity and clinical assessment by means of inclinometers (36), but also the addition of MRI follow-up before and after PRP injection. Furthermore, there was the advantage of using an apheresis machine that made it possible to standardize the PRP being used with a consistent platelet concentration (850,000 platelets/microlitre) and containing no leucocytes (leucocyte-poor PRP) (26).

One limitation of this study is the lack of a control group and the small number of participants, which can be attributed to stringent inclusion and exclusion criteria and the impact of the COVID-19 pandemic. Furthermore, the study’s follow-up evaluation was conducted only once at 1 year without any intermediate assessments. Additionally, there is a lack of information on other treatment modalities utilized by the participants during the study period. To address these limitations and provide more comprehensive insights, future research endeavours should consider incorporating a larger sample size, incorporating a control group and conducting evaluations at multiple time points. Additionally, it is crucial to acknowledge the multifactorial nature of chronic LBP, which can be influenced by various psychological, family, professional, psychiatric and contextual factors (37). Therefore, further investigations should specify the clinical profile of patients with the best response to the PRP injection in intervertebral discs and determine optimal practical conditions for the injection and post-injection rehabilitation.

In conclusion, this pilot study underscores the feasibility, safety profile and potential benefits of PRP injection for chronic LBP. While certain patients exhibited notable improvements, additional research is warranted to confirm these results and to comprehensively understand the factors influencing treatment efficacy and to optimize the application of PRP therapy in managing chronic LBP. The findings not only add to the evolving evidence base supporting the use of PRP injection but also emphasize the importance of prioritizing patient safety in exploring novel therapeutic interventions for chronic LBP.
